# Lesser-Evil Justifications: A Reply to Frowe

**DOI:** 10.1007/s10982-022-09454-w

**Published:** 2022-08-04

**Authors:** Kerah Gordon-Solmon, Theron Pummer

**Affiliations:** 1 Department of Philosophy, Queen’s University, Kingston, ON K7L 3N6 Canada; 2 Department of Philosophy, University of St Andrews, St Andrews, KY16 9AL Fife UK

## Abstract

Sometimes one can prevent harm only by contravening rights. If the harm one can prevent is great enough, compared to the stringency of the opposing rights, then one has a lesser-evil justification to contravene the rights. Non-consequentialist orthodoxy holds that, most of the time, lesser-evil justifications add to agents’ permissible options without taking any away. Helen Frowe rejects this view. She claims that, almost always, agents must act on their lesser-evil justifications. Our primary task is to refute Frowe’s flagship argument. Secondarily, it is to sketch a positive case for nonconsequentialist orthodoxy.

Sometimes one can prevent harm only by contravening rights. If the harm one can prevent is great enough, compared to the stringency of the opposing rights, then one has a *lesser-evil justification* to contravene the rights. The classic illustration is:

*Trolley*: A runaway trolley is heading to where it will kill five people. Pedestrian is standing next to a switch that will divert the trolley down a sidetrack, saving the five. However, the trolley will then kill Workman, who is trapped on the sidetrack.^1^

Most nonconsequentialists believe *Trolley* belongs to a large family of cases in which we have lesser-evil justifications that permit, but do not obligate us to contravene rights. This belief is so widely held that we will refer to it as non-consequentialist orthodoxy. In a recent article, Helen Frowe challenges this orthodoxy.^2^ She claims that in virtually all cases, including *Trolley*, if one has a lesser-evil justification to contravene rights, one *must* act on it. We will first critique her argument, then sketch a positive case for orthodoxy.

The first, flagship premise of Frowe’s argument is:

*Preventing Harm*: One is required to minimize harm to others when one can do so without violating (impermissibly contravening) anyone’s rights, and without bearing an unreasonable cost.^3^

Frowe’s second premise is that, by flipping the switch, Pedestrian would minimize harm to others without violating anyone’s rights. Her third premise is that Pedestrian can flip the switch without bearing an unreasonable cost. It follows from these premises that Pedestrian is required to flip the switch.

Some will reject Frowe’s second premise, on the grounds that flipping the switch would violate Workman’s rights.^4^ Others will reject Frowe’s third premise, on the grounds that to flip the switch would be to *make oneself a killer*, and that this is itself an unreasonable cost to force a person to bear.^5^ Still others might reject her underlying assumption that the moral reasons for preventing harm and against contravening rights can be weighed against each other. But we agree with Frowe on all these points.^6^ What we reject is *Preventing Harm* (Frowe’s first premise).

Our negative argument is drawn directly from Frowe’s own remarks. Frowe says that sometimes an agent’s moral reasons to minimize harm and her moral reasons not to contravene rights will be ‘exactly balanced’, or equally weighty. In such cases, the agent is permitted either to minimize harm or to refrain.^7^ These cases technically falsify *Preventing Harm*: they are cases in which minimizing harm is not required, even though one could do so without violating rights or incurring an unreasonable cost.

Presumably, Frowe would respond that these counterexamples to *Preventing Harm* are merely a narrow band of exceptions.^8^ To illustrate, suppose the moral reasons for and against flipping the switch in *Trolley* exactly balance, so that Pedestrian is permitted to do either. We can upset this balance simply by increasing the amount of harm flipping the switch would prevent by a relatively small amount. If, for example, by flipping the switch, Pedestrian would save five lives plus an additional person’s leg, the reasons to flip the switch would outweigh the reasons not to, and she would be required to flip it. Since orthodox nonconsequentialists hold that there is a *significant* range of cases in which one is permitted either to minimize harm or to refrain – i.e., that Pedestrian would be permitted but not required to flip the switch whether it’s to save five, six, or seven lives – Frowe can admit the relevant exceptions to *Preventing Harm* without losing dialectical ground.

The preceding reveals two crucial things. First, Frowe is not genuinely committed to *Preventing Harm*, but instead to:

*Preventing Harm (Emended)*: One is required to minimize harm to others when the moral reasons to do it outweigh the moral reasons to refrain, and one can do it without bearing an unreasonable cost.

Second, Frowe assumes that whenever the moral reasons to minimize harm do not outweigh the moral reasons to refrain, it is because the latter outweigh the former, or because the two exactly balance. But she does not argue for this assumption. In the space that remains, we show, first, how it is plausible to reject this assumption, and second, how rejecting it – while retaining *Preventing Harm (Emended)* – helps defenders of non-consequentialist orthodoxy.

*Contra* Frowe’s assumption, we propose that in all the cases in which the moral reasons to minimize harm neither outweigh, nor are outweighed by the rights-based moral reasons to refrain, the two kinds of reasons will not exactly balance but *roughly* balance. Formally, ‘roughly balanced’ means neither set of moral reasons outweighs the other and they are not exactly balanced. Substantively, it has been variously interpreted as incommensurable, indeterminate, or on a par (in Ruth Chang’s sense of ‘parity’). For our present purpose, any of these three alternatives will do.^9^

To illustrate the phenomenon, imagine, first, that one is choosing between job-offers from two different corporate law firms. The firms’ size, culture, and client rosters (etc.) are all exactly similar, but one offers a slightly higher starting salary than the other. Bracketing that pay difference, one would have no more reason to choose either firm than the other. Factoring it in, one has more reason to choose the better-paying firm. The small difference in salary breaks the stalemate. Now imagine one is choosing between a corporate law career and a philosophy career. Corporate law is more lucrative and provides greater stability; philosophy is more intellectually rewarding and allows for greater autonomy. Suppose one is genuinely torn: one finds no more reason to choose one of these careers rather than the other. Here, if one of the options were to offer a slightly higher or lower salary, it would not break the stalemate. The modest change along one dimension of one of the options does not alter the overall balance of reasons. Such ‘insensitivity to sweetening’ is a hallmark of rough balancing: the reasons to take law career A roughly balance the reasons to take the philosophy career, the reasons to take law career A+ outweigh the reasons to take law career A (because A+ pays more), and yet the reasons to take law career A+ roughly balance the reasons to take the philosophy career. By contrast, wherever reasons for and against competing alternatives *exactly* balance, insensitivity to sweetening is impossible – small improvements are decisive.^10^

Is the choice in *Trolley* between minimizing harm and contravening rights *more like* the choice between which of two corporate law firms to work at, or the choice between corporate law and philosophy? We submit that it is more like the latter, paradigm instance of rough balancing. The contrary claim, namely, that the moral reason to refrain from contravening one person’s rights against being killed as a side effect (say) *has the exact same weight* as the moral reason to save N lives, is hardly intuitive. It cries out for supporting argumentation – at minimum, for *a reason* to accept it – which is absent from Frowe’s article. Our rough balancing proposal has at least as much direct intuitive plausibility; for anyone inclined toward the orthodox view, it also has the advantage in reflective equilibrium: it supports lesser-evil permissions without requirements not only in *Trolley,* but in a range of lesser-evils cases.^11^

Accepting rough balancing gives the defender of non-consequentialist orthodoxy all they need to successfully rebut Frowe. They can claim that, compared to the strong moral reason not to contravene Workman’s right not to be killed – keep in mind, here, that rights not to be killed are among our most stringent – the moral reason to save an additional life is not always a decisive sweetener. The moral reasons to save five roughly balance the moral reason not to kill Workman, but so too do the moral reasons to save six.

When the number of lives Pedestrian can save in *Trolley* is low enough (certainly one is low enough), the moral reasons not to contravene Workman’s right not to be killed outweigh the moral reasons to minimize harm. In these cases, there is no lesser-evil justification, and it is impermissible to flip the switch. When the number of lives Pedestrian can save is higher (say, between three and eighteen), the moral reasons not to contravene Workman’s right not to be killed roughly balance the moral reasons to minimize harm. In these cases, *contra* Frowe, it is permissible to flip the switch and permissible not to. And when the number of lives Pedestrian can save is high enough (one hundred ought to do it), the moral reasons to minimize harm outweigh the moral reasons not to contravene Workman’s right not to be killed. In these cases, it is required to flip the switch.

Let us summarize. According to non-consequentialist orthodoxy, there is a significant range of cases in which one is not required to act on lesser-evil justifications. Orthodoxy is supported by *Preventing Harm (Emended)* together with the plausible claim that, in a significant range of cases (like *Trolley*), the moral reasons to minimize harm and the moral reasons not to contravene the right not to be killed roughly balance each other. At the very least, we hope to have shown that our proposal is *no less* plausible than Frowe’s. This remains dialectically significant for the defender of orthodoxy, since (again) Frowe does not argue against our proposal, nor can such an argument be extrapolated from her article.

Some proponents of non-consequentialist orthodoxy might claim that the range of cases in which we are intuitively permitted but not required to act on lesser-evil justifications is wider than the range delivered by rough balancing. Their task, presumably, would be to refute *Preventing Harm (Emended)*.^12^ Our aim has been to show that, even granting *Preventing Harm (Emended)*, the range of lesser-evil mere permissions delivered by rough balancing is significant – wide enough to belie Frowe’s view that it’s permissible not to act on lesser-evil justifications only in extremely rare cases.

We would like to end by emphasizing a further advantage of our proposal: it can accommodate the plausible thought that there will be narrower or wider ranges of cases in which one is not required to act on lesser-evil justifications, depending on the stringency of the rights at stake.

To see this, consider:

*Push*: A runaway trolley is heading to where it will kill five people. Pedestrian can push Workman in front of the trolley, saving the five. However, this will kill Workman.

We assume that there can be lesser-evil justifications to contravene rights not to be killed as a means, but that they are harder to come by. We take it that the right not to be killed as a means is more stringent than the right not to be killed as a side effect, and that there is accordingly stronger moral reason against contravening the former. For example, there is not a lesser-evil justification to push Workman in front of the trolley. Saving five lives is enough to make it permissible to contravene the right not to be killed as a side effect, but it is not enough to make it permissible to contravene the right not to be killed as a means. If, however, pushing Workman were the only way to save many more lives, this could make it permissible for Pedestrian to do so. Suppose that it is impermissible for Pedestrian to push Workman if the number saved is under 100, but permissible to do so if the number is at least 100. It is plausible that Pedestrian is also not required to push Workman, as long as the number saved is under 200. At least within this range, the moral reasons to save lives and the moral reasons not to contravene Workman’s right are roughly balanced. If the number saved is sufficiently great (say, 500), Pedestrian would be required to push Workman.

Our proposal can capture these claims by, for example, appealing to the following multiplicative model. If saving at least N lives can make it permissible to contravene one right, then saving more than [M times N] lives can make it required to do so (where M > 1). But N will be greater in the case of more stringent rights, making the difference between N and [M times N] correspondingly greater. For illustration, suppose M = 5. Then in *Trolley*, it may be that N = 3, so that Pedestrian is permitted but not required to push Workman when this saves between 3 and 15 lives. But in *Push*, it may be that N = 100, so that Pedestrian is permitted but not required to push Workman when this saves between 100 and 500 lives. The resulting picture is one in which there is a wider range of cases in which one is not required to act on lesser-evil justifications, when more stringent rights are at stake.

## Footnotes

1 Helen Frowe, ‘Lesser-Evil Justifications for Harming: Why We’re Required to Turn the Trolley’, *Philosophical Quarterly* 68 (2018): pp. 460–480, p. 461.

2 Frowe, ‘Lesser-Evil Justifications for Harming’.

3 Frowe, *Ibid.*, p. 463. We have reworded the principle slightly. For textual support for our substitutions, see Frowe, *Ibid.*, p. 461, p. 464, and p. 468.

4 *See* Judith Jarvis Thomson, ‘Turning the Trolley’, *Philosophy and Public Affairs* 36 (2008): pp. 359–374.

5 This is the view advanced by Alec Walen and David Wasserman, ‘Agents, Impartiality and the Priority of Claims Over Duties: Diagnosing Why Thomson Still Gets the Trolley Problem Wrong by Appeal to the “Mechanics of Claims”’, *Journal of Moral Philosophy* 9 (2012): pp. 545–571. Frowe targets this view.

6 For a bit more in support of Frowe’s third premise *see* Kerah Gordon-Solmon, ‘How (and How Not) to Defend Lesser-Evil Options’, *Journal of Moral Philosophy* (2022). 10.1163/17455243-20223735

7 Frowe, ‘Lesser-Evil Justifications for Harming’, p. 462 and p. 467.

8 Frowe, *Ibid.*, p. 462.

9 *See* Ruth Chang, ‘Value Incomparability and Incommensurability’, in *Oxford Handbook of Value Theory*, ed. Iwao Hirose and Jonas Olson (Oxford: Oxford University Press, 2014).

10 *See* Derek Parfit, ‘Can We Avoid the Repugnant Conclusion?’ *Theoria* 82 (2016): pp. 110–127; Ruth Chang, ‘Parity, Imprecise Comparability and the Repugnant Conclusion’, *Theoria* 82 (2016): pp. 182–214.

11 For further elaboration and defense of ‘rough balancing’ accounts of lesser-evil permissions, *see* Gordon-Solmon, ‘How (and How Not) to Defend Lesser-Evil Options’. *See also* Jonathan Quong, ‘The Permissibility of Lesser Evil’ (ms).

12 For one way to do so, *see* Gordon-Solmon, ‘How (and How Not) to Defend Lesser-Evil Options’. For a different kind of defense of lesser-evil mere permissions, we might appeal to the distinction between *requiring* moral reasons and *justifying* moral reasons; *see* Joshua Gert, ‘Requiring and Justifying: Two Dimensions of Normative Strength’, *Erkenntnis* 59 (2003): pp. 5–36. While requiring reasons contribute to it being the case that an act is required, merely justifying reasons contribute to it being the case that acts are permissible (when they would have been impermissible otherwise) without contributing to it being the case that they are required. Thus it might be that, in a range of cases, the requiring reasons not to contravene rights outweigh the requiring reasons to minimize harm, but there are also sufficiently strong justifying reasons to minimize harm.

